# Acute myocarditis during the COVID-19 pandemic: A single center experience

**DOI:** 10.1016/j.ahjo.2021.100030

**Published:** 2021-06-24

**Authors:** Matthew Petersen, Borna Mehrad, Ellen C. Keeley

**Affiliations:** a Florida Department of Medicine, University of Florida, Gainesville, FL, United States of America; b Division of Pulmonary Critical Care and Sleep Medicine, University of Florida, Gainesville, FL, United States of America; c Division of Cardiology, University of Florida, Gainesville, FL, United States of America

**Keywords:** Myocarditis, SARS-CoV-2, COVID-19

## Abstract

**Objective:**

We sought to compare the occurrence and characteristics of patients with acute myocarditis admitted during the coronavirus disease 2019 pandemic to those admitted prior.

**Design:**

We performed a retrospective chart review of patients with the primary discharge diagnosis of acute myocarditis from September 1st 2017 through August 31st 2020.

**Results:**

We identified 67 patients, 45 (67%) admitted pre-pandemic, and 22 (33%) during the pandemic. Rate of admissions for acute myocarditis was 1.5/month [95% CI 1.04–1.95] pre-pandemic, and 3.7/month [95% CI 2.36–4.97] (p < 0.001) during the first 6 months of the pandemic. Of the 22 patients admitted during the pandemic, 10 (45%) tested positive for SARS-CoV-2. Patients who tested positive for SARS-CoV-2 were older and had lower peak troponin levels.

**Conclusions:**

During the pandemic, less than half of the patients admitted with acute myocarditis tested positive for SARS-CoV-2. Patients who tested positive were older and had lower peak troponin levels.

## Highlights

• We found an increase in the occurrence of acute myocarditis during the COVID-19 pandemic
• Less than half of acute myocarditis patients tested positive for SARS-CoV-2
• SARS-CoV-2 positive patients were older and had lower peak troponin levels

## 1 Introduction

The development of myocarditis as a complication of coronavirus disease 2019 (COVID-19) has been recognized since early in the pandemic [1,2]. We sought to compare the occurrence of acute myocarditis and characteristics of patients with acute myocarditis admitted to our hospital during the COVID-19 pandemic to those admitted prior to the pandemic.

## 2 Methods

After Investigation Review Board approval, we collected data regarding admissions for the diagnosis of acute myocarditis from September 1st 2017 through August 31st 2020 from the University of Florida Health Integrated Data Repository. We reviewed all charts to verify the diagnosis of acute myocarditis using the European Society of Cardiology Working Group definition of clinically suspected myocarditis [3], and excluded all patients with troponin elevation secondary to other conditions including acute myocardial infarction (type 1 and type 2), sepsis, Takotsubo syndrome, and pulmonary embolism. We collected information regarding demographics, comorbidities, cardiac testing, laboratory values, dates of admission, treatment, and in-hospital mortality rates. We compared variables before and during the pandemic, and between SARS-CoV-2 positive and negative patients. Categorial variables were summarized as counts and percentages and were compared using chi-square testing. A 2-tailed p value of <0.05 was considered significant. All analyses were performed using Graphpad Prism (Version 7.0).

## 3 Results

We identified a total of 166 patients in the Integrated Data Repository admitted with a diagnosis code of acute myocarditis between September 1st 2017 through August 31st 2020. Following thorough chart review, 67 patients were confirmed to have a primary discharge diagnosis of acute myocarditis with no other causes for troponin elevation, including type I and type II non-ST elevation myocardial infarction and Takotsubo syndrome. These 67 patients were included in the analysis.

**Fig. 1:**
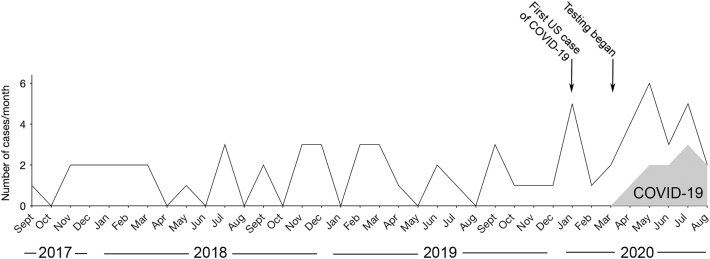
Acute myocarditis admissions from September 1, 2017 through August 31, 2020 at the University of Florida. The first case of confirmed COVID-19 in the U.S. was January 15, 2020. University of Florida started routine testing for SARS-CoV-2 in all admitted patients in March 2020 and first positive case at the University of Florida was on March 13, 2020. Acute myocarditis patients diagnosed with COVID-19 are in the shaded area.

The average rate of hospital admissions for myocarditis at the University of Florida was 1.5/month [95% CI 1.04–1.95] in the pre-pandemic months (September 1st 2017–February 29th 2020) compared to 3.7/month [95% CI 2.36–4.97] during the first 6 months of the pandemic (March 1st 2020 – August 31st 2020; p < 0.001) (Fig. 1). The first case of confirmed COVID-19 at the University of Florida was on March 13th 2020. From March 13th 2020 through August 31st 2020, a total of 1112 patients were admitted with COVID-19 to our institution.

Those who tested positive for SARS-CoV-2 were significantly older and had lower levels of high sensitivity troponin than those who tested negative for SARS-CoV-2 (Table 1). Other than these factors, we found no other significant differences between patients with acute myocarditis who presented pre-pandemic and during the pandemic (Table 1). Among the 10 patients with acute myocarditis who tested positive for SARS-CoV-2, mean symptom onset to diagnosis was 5 days [range 1–7], and their treatment included tocilizumab (n = 1), remdesivir (n = 1), steroids (n = 4), beta-blocker (n = 5), angiotensin-converting enzyme inhibitor (n = 3), anticoagulation (n = 7), and placement of a mechanical support device in one patient with cardiogenic shock. Pre-pandemic, a total of 5 patients (11%) patients underwent endomyocardial biopsy for definitive diagnosis [eosinophilic (n = 1), coxsackie (n = 1), giant cell (n = 1), fulminant lymphocytic (n = 1), presumed viral with myocyte enlargement, vacuolization and inflammation (n = 1)]. During the pandemic 1 patient (5%) underwent endomyocardial biopsy and was presumed viral (edema and inflammation present).

**Table 1:** Myocarditis before and during the COVID-19 pandemic and according to SARS-CoV-2 status.

Variable	Pre-pandemic N = 45	Pandemic N = 22	SARS-CoV-2 (−) N = 57	SARS-CoV-2 (+) N = 10	p value^c^	p value^d^
Female sex	22 (49%)	10 (45%)	28 (49%)	4 (40%)	0.85	0.97
Age (years)	40 [19–89]	49 [22–103]^a^	36 [27–49]	53 [44–72]	0.17	0.02
Hypertension	19 (42%)	9 (41%)	23 (40%)	5 (50%)	0.99	0.91
Diabetes mellitus	9 (20%)	4 (18%)	10 (19%)	3 (30%)	0.97	0.87
Hyperlipidemia	10 (22%)	4 (18%)	11 (21%)	3 (30%)	0.95	0.90
Coronary artery disease	6 (13%)	2 (9%)	7 (13%)	1 (10%)	0.93	0.95
Chronic kidney disease	5 (11%)	2 (9%)	5 (9%)	2 (20%)	0.96	0.84
Chronic obstructive pulmonary disease	6 (13%)	5 (23%)	9 (17%)	2 (20%)	0.87	0.96
Heart failure	6 (13%)	4 (18%)	8 (15%)	2 (20%)	0.93	0.93
Body mass index ≥ 30	15 (33%)	8 (36%)	19 (37%)	4 (25%)	0.97	0.87
High sensitivity troponin (pg/mL)	1360 [271–3235]	1130 [130–3279]	1694.5 [628–6161]	164 [35–278]	0.78	0.004
Brain natriuretic peptide (pg/mL)	453 [96–1291]	184 [67–882]	195 [88–1262]	206 [77–1153]	0.52	0.99
C-reactive protein (mg/L)	60 [21–129]	18 [9–177]	51.2 [10–118]	186 [113–210]	0.73	0.08
Echocardiographic/CMR abnormalities^b^	21 (47%)	13 (62%)	27 (47%)	5 (50%)	0.94	0.82
Viral etiology	28 (62%)	15 (68%)	33 (58%)	10 (100%)	0.95	0.74
Idiopathic etiology	12 (27%)	5 (22%)	17 (30%)	0 (0%)	0.94	0.61
Symptom onset to diagnosis (days)	4 [2–6]	3.5 [1–6]	3 [1–5]	5 [5–7]	0.11	0.35
Length of stay (days)	5 [3–10]	9 [4–13]	5 [3–5]	10.5 [9–11]	0.2	0.11
Cardiogenic shock	6 (13%)	3 (13%)	7 (12%)	2 (10%)	1.0	0.93
In-hospital mortality	1 (2%)	0	0	0	0.88	1.0
Ventricular arrhythmias	6 (13%)	1 (5%)	7 (12%)	0	0.85	0.75
Atrial arrhythmias	4 (9%)	1 (5%)	4 (7%)	1 (10%)	0.91	0.91

Data presented as N (%), median with 25–75 interquartile range. CMR = cardiac magnetic resonance.

a One patient was 103 years old.

b Wall motion abnormalities, newly depressed left ventricular systolic function on echocardiography, evidence of late gadolinium enhancement and/or edema on CMR.

c Comparison between pre-pandemic and pandemic.

d Comparison between SARS-CoV-2 (+) and SARS-CoV-2 (−).

## 4 Discussion

We found a two-fold increase in the occurrence of acute myocarditis admissions during the first 6 months affected by the COVID-19 pandemic compared to years past. In addition, patients admitted with acute myocarditis during the pandemic who tested positive for SARS-CoV-2 were significantly older and had lower levels of high sensitivity troponin than those who tested negative. Less than half of the patients admitted with myocarditis during the early months of the pandemic, however, tested positive for SARS-CoV-2. While all SARS-CoV-2 positive patients were diagnosed with traditional cardiotropic viruses known to cause myocarditis including adenovirus, cytomegalovirus, enterovirus and parvovirus, it is possible that these patients had false negative testing for SARS-CoV-2, or a delayed COVID-19 presentation such that the infection had already cleared. High rates of co-infection between SARS-CoV-2 and other respiratory pathogens [4] and delayed presentation of myocarditis due to COVID-19 in patients who test negative for SARS-CoV-2 on admission have been recently reported [5,6].

Despite multiple reports of COVID-19 related myocarditis cases [2], recent pathological studies suggest that direct SARS-CoV-2 infiltration into the myocardium is exceedingly rare. In these studies, investigators reviewed endomyocardial biopsy and autopsy series and found that COVID-19 related myocarditis occurred in ~4.5% of patients [7,8]. Moreover, due to the referral bias for endomyocardial biopsy and autopsy, they suggest the true incidence is likely even lower. These studies underscore the need for a more standardized approach in reporting cardiac pathologic findings in COVID-19, such as myocarditis, and question the utility for routine endomyocardial biopsy testing considering the low yield and risks associated with this procedure.

Limitations of our study include the fact that it is a small, single-center, retrospective study. Diagnosis of acute myocarditis was made on the basis of a combination of non-invasive testing (including CMR), endomyocardial biopsy, virology testing and high clinical suspicion. While not all patients underwent endomyocardial biopsy or CMR imaging, they were deemed to have clinically suspected myocarditis according to previously published guidelines [3], and our thorough chart review confirmed that acute myocarditis was the primary discharge diagnosis in these 67 patients. It is possible that the observed increase in acute myocarditis cases is related to a more investigative approach by the medical team during the pandemic searching for an association with SARS-CoV-2. Lastly, antibody testing for prior SARS-CoV-2 infection in patients who were SARS-CoV-2 negative on admission was not routinely performed as part of standard clinical care at our institution.

In conclusion, we found a two-fold increase in occurrence of acute myocarditis admissions during the first 6 months affected by the COVID-19 pandemic compared to years past. Less than half of the patients tested positive for SARS-CoV-2. Patients who tested SARS-CoV-2 positive were older and had lower peak levels of high sensitivity troponin compared to those who tested negative for the virus.

## Sources of funding

None.

## Declaration of competing interest

No authors reported conflict of interest.
